# Impact of community-acquired paediatric rotavirus gastroenteritis on family life: data from the REVEAL study

**DOI:** 10.1186/1471-2296-11-22

**Published:** 2010-03-15

**Authors:** Marie Van der Wielen, Carlo Giaquinto, Leif Gothefors, Christel Huelsse, Frédéric Huet, Martina Littmann, Melanie Maxwell, José MP Talayero, Peter Todd, Miguel T Vila, Luigi Cantarutti, Pierre Van Damme

**Affiliations:** 1 The Centre for the Evaluation of Vaccination, Vaccine and Infectious Disease Institute, University of Antwerp, Belgium; 2 Department of Pediatrics, University of Padua, Italy; 3 Department of Clinical Sciences/Pediatrics, Umeå University, Sweden; 4 Landesgesundheitsamt, Rostock, Germany; 5 Pediatric Service 1, Hôpital d'Enfants, Dijon, France; 6 Wirral Hospital NHS Trust, Wirral, Merseyside, UK; 7 Pediatrics Department, Hospital Marina Alta, Denia, Spain; 8 Pediatrics Department, Hospital Francesc de Borja, Gandía, Spain; 9 Pedianet Network, Italy

## Abstract

**Background:**

Rotavirus is the leading cause of acute gastroenteritis (AGE) and the most frequent cause of severe diarrhoea in children aged less than 5 years. Although the epidemiology of rotavirus gastroenteritis (RVGE) is well documented, there are few data on the impact of RVGE on the families of affected children.

**Methods:**

Data associated with the burden of RVGE, including number of working days lost, levels of parental stress, the need for alternative childcare arrangements and additional nappies used, were extracted from questionnaires completed by parents of children participating in a prospective, multicentre, observational study (Rotavirus gastroenteritis Epidemiology and Viral types in Europe Accounting for Losses in public health and society, REVEAL), conducted during 2004-2005 in selected areas of Belgium, France, Germany, Italy, Spain, Sweden, and the United Kingdom to estimate the incidence of RVGE in children aged less than 5 years seeking medical care as a result of AGE.

**Results:**

1102 children with RVGE were included in the present analysis. The proportion of RVGE cases that required at least one parent or other person to be absent from work was 39%-91% in the hospital setting, 44%-64% in the emergency department, and 20%-64% in primary care. Self-reported levels of parental stress were generally high (mean stress levels, ≥ 5 on a 10-point visual analogue scale). Additional childcare arrangements were required in up to 21% of RVGE episodes. The mean number of nappies used per day during RVGE episodes was approximately double that used when the child was not ill.

**Conclusions:**

Paediatric RVGE cases cause disruption to families and parental stress. The burden of RVGE on children and their families could be substantially reduced by routine rotavirus vaccination of infants.

## Background

Rotavirus is the leading cause of acute gastroenteritis (AGE) and the most frequent cause of severe diarrhoea in children aged less than 5 years [1,2]. Although the epidemiology of and health-care costs associated with RVGE are well documented, there are few data on the impact of RVGE on the families of affected children.

It is estimated that RVGE is the cause of 611,000 deaths annually in children aged less than 5 years [3], of which approximately 230 occur in the European Union [4]. Although mortality due to RVGE is relatively low in industrialized regions, diarrhoea remains a common cause of physician visits and hospital admissions in children aged less than 5 years [1,5]. Moreover, the peak incidence of RVGE occurs between January and March [2,6,7], which coincides with the peak incidences of other infections, such as respiratory-tract infections caused by respiratory syncytial virus or influenza virus. The high rate of hospitalisation associated with RVGE at this time of year can place a considerable additional burden on hospital services [8].

The Rotavirus gastroenteritis Epidemiology and Viral types in Europe Accounting for Losses in public health and society (REVEAL) study is a prospective, 1-year observational cohort study that was conducted between October 2004 and September 2005 using a common protocol in selected areas of Belgium, France, Germany, Italy, Spain, Sweden and the UK. The aims of this study were to determine the incidence and burden of RVGE in children aged less than 5 years with AGE and seeking medical attention in a primary-care, emergency or hospital setting. Full details of the study design and sampling procedures have been reported previously [2]. The study also described the distribution of rotavirus serotypes [9], the clinical impact [10] and the economic and societal costs of RVGE in these seven countries [11]. A total of 2846 children with AGE were included in the study. The number of children included in each study area varied from 127 children in Belgium to 801 children in Spain; the majority of the children in all countries and settings were younger than 2 years of age [2].

The results of tests for rotavirus by enzyme-linked immunosorbent assay (ELISA) were available for 2712 children. Overall, ELISA results were positive for 1102 children (40.6%) [9], ranging from 33% in Germany and Spain to 59% in Sweden. The percentages of children with AGE who were found to be rotavirus positive ranged from 53% in Spain to 69% in Italy for hospitalised children, from 35% in Spain to 64% in Sweden for those seen in emergency departments, and from 7% in Sweden to 41% in Belgium for those seen in primary-care settings.

Morbidity associated with this illness has been shown to be a substantial burden for children, health-care providers and society in Europe [10-13]. Here we report data on the burden of paediatric community-acquired RVGE on family life, collected as part of the REVEAL study.

## Methods

We analysed data collected during the REVEAL study. Full details of this prospective, multicenter, study, including design and sampling procedures, have been reported previously [2]. In brief, the REVEAL Study was an observational study of AGE in children <5 years of age in selected areas of 7 European countries. Data were collected and analyzed for each country for a 12-month period. The study determined the annual incidence rates for AGE and RVGE in children <5 years of age in primary care, emergency department, and hospital settings in a specific study area in each country, using the same protocol. Children were excluded if no written consent was obtained or if they were not native speakers of the language spoken in the country or if they had no telephone access. The study was conducted in accordance with the 2004 amendment of the Declaration of Helsinki, the guidelines for Good Epidemiological Practice [14], and local regulatory requirements. The protocol was approved by the local ethics committee in each study area.

The present study included all children from the REVEAL study with ELISA-confirmed RVGE (n = 1102). This included 57 children from the study areas in Belgium, 99 from France, 158 from Germany, 336 from Italy, 252 from Spain, 124 from Sweden, and 76 from the UK. Parents of the participating children completed a follow-up questionnaire relating to parameters associated with the burden of AGE, including: the number of working days lost by parents or other persons; the need for alternative childcare arrangements (e.g. baby sitters, other family members); the number of nappies usually used when the child was healthy and the total number of nappies used from the onset of the illness to the end of symptoms (excluding any time the child was in hospital); and self-reported levels of stress experienced by parents during the illness, with the intensity of stress ranked on a 10-point visual analogue scale where 1 = no stress experienced and 10 = felt extremely stressed [2]. The parents were required to return the completed questionnaire to the investigator within 14 days after the onset of symptoms of AGE.

Statistical analyses were performed using SAS software (version 8.2; SAS Institute).

## Results

The overall response rate in the REVEAL study was generally very high; 98% of follow-up questionnaires were completed and retrieved from parents of children with AGE in the selected regions of France (274/281), 89% in the German regions (443/498), 97% in Italy (731/757), 93% in Spain (740/799), and 89% in Sweden (202/220). However, lower response rates were seen in the UK study areas (102/160; 64%) and Belgium (82/127; 65%).

### Working days lost

Across all settings and study areas, the proportion of RVGE cases that required at least one parent or other person to be absent from work ranged from 39% to 91% of cases in the hospital setting, from 44% to 64% of cases in the emergency-department setting, and from 20% to 64% of cases in the primary-care setting (Table 1). The countries in which a person other than the child's parents was most likely to lose time from work were Belgium, Spain and the UK.

**Table 1:** Percentage of cases of rotavirus-positive gastroenteritis (RVGE) that required at least one parent or other person to be absent from work

Study area	Cases of RVGE requiring at least one parent or other person to be absent from work					
	**Hospital**		**Emergency department**		**Primary care**	
	
	**%**	***n/N***	**%**	***n/N***	**%**	***n/N***

**Belgium**	39	11/29	50	1/2	64	9/14
**France**	43	13/30	44	22/50	42	8/19
**Germany**	53	17/32	--^a^	--^a^	36	36/99
**Italy**	81	43/55	64	94/147	44	55/125
**Spain**	68	30/46	44	41/96	32	30/94
**Sweden**	73	46/63	58	29/51	100^b^	1/1^b^
**United Kingdom**	91	20/22	50	8/16	20	2/10

^a^In the German study area, all eligible children who presented to the emergency department with RVGE during the study were referred to the hospital, so there were no inclusions for the emergency department setting.

^b^Only one child with RVGE was included from the primary-care setting in Sweden; both parents lost at least 1 day from work.

The mean number of working days lost by parents or other persons caring for children with RVGE ranged from 2.3 days (hospital, France) to 7.5 days (primary care, UK) (Table 2). In general, mothers were absent from work for approximately twice as long as fathers (data not shown).

**Table 2:** Mean number of working days lost by parents or another person due to their child's rotavirus-positive gastroenteritis^a^

Study area	Mean number of working days lost (standard deviation)		
	
	Hospitalised cases	Emergency cases	Primary-care cases
**Belgium**	4.2 (2.9)	4.00	4.8 (4.9)
**France**	2.3 (2.7)	2.5 (1.5)	3.4 (2.1)
**Germany**	6.4 (2.5)	---^b^	5.3 (2.9)
**Italy**	5.4 (3.8)	3.8 (2.8)	3.7 (2.6)
**Spain**	4.6 (2.2)	4.4 (4.5)	4.1 (3.4)
**Sweden**	3.8 (2.6)	4.3 (3.0)	5.0^c^
**United Kingdom**	4.0 (2.7)	2.9 (3.0)	7.5 (6.4)

^a^The data given in this table are derived from the same population as in Table 1, and thus the denominators '*N*' for each setting are the same as given in Table 1.

^b^In Germany, all eligible children who presented to the emergency department with RVGE during the study were referred to the hospital, so there were no inclusions for the emergency-department setting.

^c ^Only one child with RVGE was included from the primary-care setting in Sweden.

### Additional childcare

Across study areas, additional childcare arrangements (e.g. because of exclusion of the child from collective childcare) were required in up to 21% of RVGE episodes (hospital, Sweden; Table 3). The need for additional childcare was identified with a general question that asked whether anyone was hired specifically to assist with care of the child during the RVGE episode. The mean duration of additional childcare ranged from 1.31 days (hospital, Sweden) to 7 days (hospital, France) (Table 3). No additional childcare was required for children with RVGE hospitalized in Spain or for those managed by the emergency department in the UK.

**Table 3:** Percentage of cases for which additional childcare was sought during episodes of rotavirus-positive gastroenteritis (RVGE) and mean duration of additional childcare^a^

Study area	Cases of RVGE requiring at least one parent or other person to be absent from work					
	**Hospital**		**Emergency department**		**Primary care**	
	
	**% of cases (*n*)**	**Mean duration (days)**	**% of cases (*n*)**	**Mean duration (days)**	**% of cases (*n*)**	**Mean duration (days)**

**Belgium**	7.4 (2)	4.00	0	0	7.1 (1)	5.00
**France**	3.3 (1)	7.0	0	0	0	0
**Germany**	0	0	--^b^	--^b^	6.1 (6)	4.08
**Italy**	4.0 (2)	3.50	5.3 (7)	2.86	8.5 (10)	3.00
**Spain**	0	0	3.3 (3)	4.83	3.2 (3)	4.67
**Sweden**	21.3 (13)	1.31	14.0 (7)	2.86	0^c^	0^c^
**United Kingdom**	14.3 (3)	3.00	0	0	11.1 (1)	3.00

^a^The data given in this table are derived from the same population as in Table 1, and thus the denominators '*N*' for each setting are the same as given in Table 1.

^b^In Germany, all eligible children who presented to the emergency department with RVGE during the study were referred to the hospital, so there were no inclusions for the emergency-department setting.

^c ^Only one child with RVGE was included from the primary-care setting in Sweden.

### Use of nappies

The mean number of nappies used per day during an episode of RVGE was approximately double that used when the child was not ill. Across all countries and settings for children with RVGE, the mean number ± standard deviation (SD) of nappies used per day ranged from between 4.22 ± 1.67 (hospital, Germany) and 5.65 ± 2.96 (hospital, Spain) when the child was healthy, to between 8.76 ± 1.95 (primary care, France) and 12.77 ± 4.72 (hospital, Spain) while the child was ill (data not shown).

### Parental stress

The self-reported levels of parental stress during the episode of RVGE were generally high. With few exceptions across settings and study areas, the mean stress levels reported by both parents were 5 or more on the 10-point visual analogue scale (Table 4). With the exception of cases managed in the emergency department in Belgium (*n *= 5), where the mean levels of stress were the same for both parents, the mean level of stress reported by mothers was higher than that for fathers (Table 4). In general, mean levels of parental stress were lower for children treated in the emergency department or in a primary-care setting than in hospital, although this was not the case for Italian mothers or Swedish fathers of children treated in the emergency department, or for Swedish parents or UK fathers of children managed in primary care (Table 4).

**Table 4:** Self-reported levels of stress experienced by parents of children with rotavirus-positive gastroenteritis; mean values and standard deviation on a visual analogue scale where 1 = no stress and 10 = extremely stressed.

Study area	Parent	Mean level of stress (standard deviation)		
		
		Hospital	Emergency department	Primary care
**Belgium**	Mother	7.44 (1.93)	4.5 (4.95)	6.57 (2.24)
	Father	6.79 (2.38)	4.5 (4.95)	6.40 (2.72)
**France**	Mother	8.27 (1.51)	7.10 (2.43)	5.82 (2.38)
	Father	6.90 (2.09)	6.09 (2.61)	5.09 (2.40)
**Germany**	Mother	8.53 (1.80)	--^a^	6.57 (2.29)
	Father	7.40 (2.85)	--^a^	5.71 (2.55)
**Italy**	Mother	7.53 (2.37)	7.71 (2.14)	5.46 (2.61)
	Father	7.25 (2.46)	6.85 (2.42)	4.83 (2.46)
**Spain**	Mother	8.99 (1.37)	8.64 (1.45)	7.18 (2.06)
	Father	8.56 (1.53)	8.31 (1.76)	6.61 (2.33)
**Sweden**	Mother	6.71 (2.23)	6.52 (2.46)	8.00^b^
	Father	5.30 (2.45)	5.77 (2.68)	7.00^b^
**United Kingdom**	Mother	8.89 (1.54)	7.44 (2.03)	8.40 (1.35)
	Father	7.45 (2.68)	6.63 (2.29)	7.75 (1.75)

^a ^In the German study area, all eligible children who presented to the emergency department with acute gastroenteritis during the study were referred to the hospital, so there were no inclusions for the emergency-department setting.

^b ^Only one child with RVGE was included from the primary-care setting in Sweden.

## Discussion

To our knowledge, the REVEAL study is the first large-scale European study to document the impact of RVGE on family life [2]. Our results suggest that RVGE places a considerable burden on the family of the affected child. In 39-91% of all RVGE cases, according to health-care setting, at least one parent was required to be absent from work. The mean number of working days lost ranged between 2 and 7 days, and approximately one quarter of episodes of RVGE required additional childcare for a mean of up to 7 days. In addition, the number of nappies used per day during episodes of RVGE was approximately double that used when the child was healthy. As might be expected, parents reported high levels of stress as a result of their child's illness.

With regard to the parameters considered in the present analysis, the data extracted from the REVEAL study do have some limitations. First, only children seeking health care were included in the study and, due to the inclusion criteria, not all cases of RVGE in the study area were included. However, we believe it is reasonable to assume that non-included cases would have similar characteristics [2]. Second, the analyses reported here rely on data collected via questionnaires that the parents completed retrospectively after the child had recovered from the RVGE episode, raising the potential for bias due to a number of different factors. For example, there may have been incomplete or inaccurate recall of the amount of time lost from work or levels of stress experienced during the child's illness. In addition, parents who felt more stressed during the episode of illness might have been more likely to complete and return the follow-up questionnaire. A further potential weakness is that the reported levels of parental stress associated with the RVGE episode were subjective assessments provided by the parents on a single occasion, although a visual analogue scale is a well-accepted tool for collecting such information. Furthermore, the exclusion criteria (no telephone access, not native speakers of local language) may have led to some bias in some results. Finally, only a small number of cases were included in some settings, and consequently the mean values reported may have been influenced by wide ranges in the observed data.

A previous analysis of data collected during the REVEAL study demonstrated the substantial economic burden of RVGE from the health-care and societal perspectives [11]. That analysis found that a considerable proportion of the societal costs were attributable to loss of productivity due to absenteeism by parents or other carers, while the child was ill. It was also clear that the loss of income caused by an absence of up to 7 working days also represented a considerable financial burden for the child's family [11]. The requirements for additional childcare and the substantial increase in the use of nappies that we have identified in the present study would further add to the costs of RVGE for the family. We have also reported previously that a substantial proportion of children with RVGE included in the REVEAL study visited more than one health-care setting during their illness [10]. Of children who first consulted in the primary-care setting, 33-68% subsequently required medical care in another clinical setting. This additional demand upon parents may represent a further logistical and financial burden.

Our results also show that paediatric RVGE caused considerable stress for parents. In general, both fathers and mothers rated their level of stress during the episode of illness as > 5 on the 10-point scale, regardless of the setting in which the child was treated (although stress among parents of children managed in hospital tended to be higher). This is consistent with the results of a small study performed in the USA, in which parents of children with RVGE managed in either the emergency department or hospital setting reported that their child's illness had caused them stress, anxiety, loss of sleep and inconvenience (absence from work, increased use of nappies) and disrupted normal family life [15].

There are very few data available regarding the impact of acute childhood illnesses on family life. However, a recent study of the burden of rotavirus diarrhoea among children attending day-care centres in Lyon, France, found that in 58% of cases of RVGE at least one parent was required to take leave of absence from work (mean, approximately 2 days) [16]. Similarly, a study of RVGE in Belgium found that parents missed a mean of 1.5 days of work for cases managed in primary care and 2.5 days for hospitalised cases [13]. These findings are consistent with observations made in the present study. To put this in context with other childhood illnesses, a study of children with acute otitis media in Israel found that the mean number of working days lost by parents was 1.6 ± 1.8 days [17] compared with means of 2 to 7 days in the present study.

## Conclusions

The REVEAL study has provided valuable information about the substantial economic burden, stress and disruption for families caused by community-acquired paediatric RVGE. As a result of RVGE, parents or other carers typically lost time from work, and additional childcare arrangements were also required for up to a quarter of cases. The use of nappies doubled during the period that the child was ill. Taken together with concern about their child, these effects undoubtedly contributed to the high levels of stress reported by parents as a result of the episode of RVGE.

The burden of RVGE on society, health-care systems, children and their families could be substantially reduced by routine rotavirus vaccination of infants.

## Competing interests

Pierre Van Damme acts as chief and principal investigator for clinical trials conducted on behalf of the University of Antwerp, for which the University obtains research grants from vaccine manufacturers; speakers fees for presentations on vaccines are paid directly to an educational fund held by the University of Antwerp. CG has received research grants and speaker's honoraria from Sanofi Pasteur MSD and GlaxoSmithKline Biologicals. LC has received research grants from Sanofi Pasteur MSD and GlaxoSmithKline Biologicals. LG has been principal investigator of vaccine trials for GlaxoSmithKline, Merck, Sanofi Pasteur MSD, Wyeth, and MedImmune. CH has received research grants and speaker's honoraria from Sanofi Pasteur MSD and GlaxoSmithKline Biologicals. ML has received research grants and speaker's honoraria from Sanofi Pasteur MSD and GlaxoSmithKline Biologicals. All other authors report no potential conflicts.

## Authors' contributions

All authors were principal investigators of the REVEAL study, and thus involved in conception, design and acquisition of data, except LC who was a participating physician in the REVEAL study and who contributed to collection of data. All authors were involved in the drafting and/or revision of this manuscript, and all have approved the final version.

## Pre-publication history

The pre-publication history for this paper can be accessed here:

http://www.biomedcentral.com/1471-2296/11/22/prepub
